# Survival time prediction in patients with high-grade serous ovarian cancer based on ^18^F-FDG PET/CT- derived inter-tumor heterogeneity metrics

**DOI:** 10.1186/s12885-024-12087-y

**Published:** 2024-03-12

**Authors:** Dianning He, Xin Zhang, Zhihui Chang, Zhaoyu Liu, Beibei Li

**Affiliations:** 1 College of Medicine and Biological Information Engineering, Northeastern University, Shenyang, China; 2 Department of General Surgery, Shengjing Hospital of China Medical University, 110004 Shenyang, P.R. China; 3 Department of Radiology, Shengjing Hospital of China Medical University, No. 36, Sanhao Street, Heping District, Shenyang, Liaoning, 110004 P.R. China

**Keywords:** High-grade serous ovarian cancer, Positron emission tomography, Computed tomography, Prognosis, Heterogeneity

## Abstract

**Background:**

The presence of heterogeneity is a significant attribute within the context of ovarian cancer. This study aimed to assess the predictive accuracy of models utilizing quantitative ^18^F-FDG PET/CT derived inter-tumor heterogeneity metrics in determining progression-free survival (PFS) and overall survival (OS) in patients diagnosed with high-grade serous ovarian cancer (HGSOC). Additionally, the study investigated the potential correlation between model risk scores and the expression levels of p53 and Ki-67.

**Methods:**

A total of 292 patients diagnosed with HGSOC were retrospectively enrolled at Shengjing Hospital of China Medical University (median age: 54 ± 9.4 years). Quantitative inter-tumor heterogeneity metrics were calculated based on conventional measurements and texture features of primary and metastatic lesions in ^18^F-FDG PET/CT. Conventional models, heterogeneity models, and integrated models were then constructed to predict PFS and OS. Spearman’s correlation coefficient (ρ) was used to evaluate the correlation between immunohistochemical scores of p53 and Ki-67 and model risk scores.

**Results:**

The C-indices of the integrated models were the highest for both PFS and OS models. The C-indices of the training set and testing set of the integrated PFS model were 0.898 (95% confidence interval [CI]: 0.881–0.914) and 0.891 (95% CI: 0.860–0.921), respectively. For the integrated OS model, the C-indices of the training set and testing set were 0.894 (95% CI: 0.871–0.917) and 0.905 (95% CI: 0.873–0.936), respectively. The integrated PFS model showed the strongest correlation with the expression levels of p53 (*ρ* = 0.859, *p* < 0.001) and Ki-67 (*ρ* = 0.829, *p* < 0.001).

**Conclusions:**

The models based on ^18^F-FDG PET/CT quantitative inter-tumor heterogeneity metrics exhibited good performance for predicting the PFS and OS of patients with HGSOC. p53 and Ki-67 expression levels were strongly correlated with the risk scores of the integrated predictive models.

**Supplementary Information:**

The online version contains supplementary material available at 10.1186/s12885-024-12087-y.

## Background

Ovarian cancer is the third most common cancer among women [1]. In 2023, there were 19,710 estimated new cases (2% of all types of cancers) and 13,270 deaths (5% of all cancer deaths) in women in the United States [2]. The 5-year relative survival rate was approximately 50% in all races and ethnicities from 2012 to 2018 [2]. High-grade serous ovarian cancer (HGSOC) is the most common and fatal subtype of ovarian cancer, accounting for 70–80% of total deaths [3, 4]. Previous studies have shown that the International Federation of Gynecology and Obstetrics (FIGO) stage, CA125 level, and tumor grade help predict the prognosis of patients with ovarian cancer [5]; however, they are not strong, independent prognostic indicators. Thus, reliable biomarkers for predicting the survival of patients with ovarian cancer are lacking [6].

Ovarian tumors are highly heterogeneous [7, 8], with intra- and inter-tumor heterogeneity. Intra-tumor heterogeneity refers to the inconsistency observed within a single lesion. Inter-tumor heterogeneity, also known as spatial heterogeneity, refers to variations in multiple lesions of the same tumor type in one patient. The existence of tumor heterogeneity may explain the rapid progression of ovarian cancer and inconsistent response to the same treatment regimen [9]. A better understanding of tumor heterogeneity may facilitate appropriate tumor stratification to achieve optimal individualized interventions [9, 10]. Hence, there is an urgent need to develop simple and noninvasive methods to assess heterogeneity and integrate these methods into clinical pathways.

Radiomics can be employed to convert traditional medical images into several high-dimensional quantitative imaging features that can be mined using computer algorithms [11]; providing a noninvasive quantitative approach to estimate tumor heterogeneity [12–14], particularly inter-tumor heterogeneity. To date, only a few studies have investigated image-based inter-tumor heterogeneity. These studies have showed that Computer Tomography (CT) -based inter-tumor heterogeneity metrics are related to survival time [15], platinum resistance [16], and response to immunotherapy [17] in patients with ovarian cancer.

Ovarian cancer, particularly HGSOC, is frequently diagnosed at an advanced stage with peritoneal implantation metastasis due to the lack of evident symptoms in the early stages [18, 19]. CT is a commonly used preoperative modality for ovarian cancer; however, its sensitivity for detecting peritoneal metastasis is relatively low [20, 21]. ^18^F-fluoro-2-deoxyglucose Positron Emission Tomography/Computed Tomography (^18^F-FDG PET/CT), which provides both anatomical location and metabolic information, is beneficial for staging malignant gynecological tumors [19, 22, 23]. Although there is some controversy regarding the effectiveness of ^18^F-FDG PET/CT for evaluating peritoneal implantation in ovarian cancer [19, 24–26], a recent meta-analysis showed that ^18^F-FDG PET/CT had inferior sensitivity but superior specificity for the detection of metastasis [20]. Nevertheless, to date, no research has specifically investigated the inter-tumor heterogeneity derived from ^18^F-FDG PET/CT.

The present study aimed to establish inter-tumor heterogeneity metrics using two modalities (PET and CT) and two dimensions (conventional measurements and texture features) of ^18^F-FDG PET/CT images. The study evaluated the performance of models based on these metrics to predict progression-free survival (PFS) and overall survival (OS) of patients with HGSOC. The correlations between the prognostic models and p53 and Ki-67 expression levels were also determined to provide reliable support for the preoperative evaluation of patients with HGSOC.

## Patients and methods

### Patient characteristics

This study was approved by the Ethics Committee of Shengjing Hospital of China Medical University (No. 2021PS881K). The requirement for informed consent was waived because of the retrospective nature of the study.

We enrolled patients who underwent preoperative ^18^F-FDG PET/CT examination and were suspected with ovarian cancer at Shengjing Hospital of China Medical University between January 1, 2010 and December 30, 2020. The inclusion criteria were as follows: (1) patients with a primary tumor, (2) patients in whom cytoreductive surgery was performed within 15 days after ^18^F-FDG PET/CT examination at our hospital, (3) patients who received no neoadjuvant chemotherapy before surgery, and (4) patients who were pathologically confirmed to have HGSOC after surgery. The exclusion criteria were as follows: (1) patients in whom the primary ovarian lesion could not be identified on ^18^F-FDG PET/CT images, (2) patients with apparent artifacts (including hip replacement and uterine contraceptive ring artifacts) in CT images that affected the observation of tumor lesions, (3) patients in whom ^18^F-FDG PET/CT images showed no metastatic lesions or metastasis could not be identified, and (4) patients with incomplete data. All patients received platinum-based chemotherapy after surgery. Patients’ clinical details, including age, CA125 level, FIGO stage, volume of ascites, characteristics of ascites, surgical resection status (Sur_status), PFS, and OS, were recorded. PFS was defined as the time between the preoperative CT scan and tumor progression, whereas OS was defined as the time from the preoperative CT examination to death [27].

### Image acquisition and segmentation

All patients underwent ^18^F-FDG PET/CT scan using Discovery PET/CT 690 (GE Healthcare, Milwaukee, USA) according to the European Association of Nuclear Medicine guidelines [28, 29]. ^18^F-fluoro-2-deoxyglucose (^18^F-FDG) was synthesized at the PET/CT center of our hospital. ^18^F-FDG [28] requires quality control with a pH of 4.5–8.5 and purity of > 98%. The patients fasted for 4–6 h, and the blood glucose limit was < 15 mg/L; the injection dose for patients was 15 mCi/kg (± 10%). Sixty minutes after the injection, patients underwent 18F-FDG PET/CT. The median applied activity of ^18^F-FDG was 310.45 MBq (1st Qu: 269.00 MBq to 3rd Qu: 354.00 MBq). The scanning region ranged from above the upper thigh to the top of the head. The tube voltage of the CT was 120 kV, automatic tube current was 15–180 mA, tube rotation speed was 0.8 s/rot, and scanning layer thickness was 3.8 mm. PET scans were performed using the three-dimensional acquisition mode, and 8–9 beds were collected, with each bed collected for 3 min. The size of the reconstruction matrix was 192 × 192 pixels. The image was reconstructed using the ordered subset maximum expectation iteration method.

Two researchers (ZDG and SBH, with 15 and 12 years of experience in interpreting ^18^F-FDG PET/CT images, respectively) evaluated the ^18^F-FDG PET/CT images and identified all suspected ovarian cancer lesions, including primary lesions and peritoneal metastatic implants in the abdominopelvic cavity. The location of the lesions was digitally encoded based on the anatomical abdominopelvic region [30, 31], as shown in Additional file 1.

Subsequently, the ^18^F-FDG PET/CT images were loaded onto the IntelliSpace Discovery platform (version 3.0, Philips Healthcare, Eindhoven, Netherlands). The “Research Oncology Suite” was used to delineate the volumes of interest (VOIs). This suite can simultaneously display the corresponding layers of CT and PET sequences, and researchers can adjust the range of VOIs on the CT sequence with metabolic information shown on PET sequences as a reference. We used the solid component of lesions with ^18^F-FDG uptake in PET to delineate VOIs. The cystic components of the lesions were mostly liquid and contained very few tumor cells, whereas the peritoneal metastatic implants were almost solid. Therefore, using the solid components of lesions for analysis better reflects the heterogeneity between tumor cells and further enhances the accuracy of the results.

The primary focus and all selected peritoneal metastatic implants (1–9 areas) were delineated for each patient. Only one lesion per area was selected. If there were multiple lesions in an area, the largest lesion was selected as the VOI. Each VOI had to be greater than 5 mm × 5 mm × 5 mm, and this process was performed according to the Image Biomarker Standardization Initiative (IBSI) [32]. To assess the reproducibility of both intra- and interobserver segmentation, two researchers (ZDG and SBH) repeated the segmentation process on 30 randomly chosen cases after a lapse of 1 month.

### Extraction of inter-tumor heterogeneity metrics based on conventional measurements

Each VOI had eight conventional measurements, including pixel number (Pixel_number), major axis length (Maj), minor axis length (Min), CT value (HU), maximum standardized uptake value (SUVmax), mean standardized uptake value (SUVmean), peak standardized uptake value (SUVpeak), and total lesion glycolysis (TLG) in the pathological area. To quantify the inter-tumor heterogeneity of conventional measurements, we calculated the following statistics for every VOI: (1) central tendency statistics: mean, median, mode, and quartile deviation (R); (2) discrete trend statistics: standard deviation (Std_dev), standard error for the sample mean (SM), variance, range, coefficient of variation (CV), corrected sum of squares (CSS), uncorrected sum of squares (USS); and (3) distribution statistics: kurtosis and skewness. Therefore, 13 qualitative heterogeneity metrics were generated. For example, patient 1 had three VOIs (one primary focus and two peritoneal metastatic implants). Each VOI had eight conventional measurements, and each conventional measurement had 13 inter-tumor heterogeneity metrics at the patient level. Taking conventional measurement SUVmean as an example, SUVmean_Mean, SUVmean_Median, SUVmean_Mode, SUVmean_td_dev, SUVmean_Variance, SUVmean_Range, SUVmean_CV, SUVmean_CSS, SUVmean_USS, SUVmean_R, SUVmean_SM, SUVmean_Skewness, and SUVmean_Kurtosis were generated. Finally, 104 heterogeneity metrics based on conventional measurements were obtained for each patient.

### Extraction of inter-tumor heterogeneity metrics based on CT texture features

Inter-tumor heterogeneity metrics based on texture features were extracted as follows: (1) CT images were rescaled and 256 Gy levels were used. Moreover, a bin width of 32 was used to discretize the images; (2) the Gray-Level Co-occurrence Matrix (GLCM) of each VOI voxel was calculated; (3) the Haralick texture features, including energy, entropy, contrast, and homogeneity, were calculated; (4) the lesions were divided into different subregions using the clustering algorithm; (5) pairwise similarities between subclasses were quantified as a dissimilarity matrix using Euclidean distance. Cluster site entropy (cSE) was calculated based on the frequency of pairwise similarities; and (6) The grey level distance zone matrix (GLDZM) was established. Cluster standard deviation (cluDev) and cluster dissimilarity (cluDiss) were calculated for GDM. More detailed information can be found in Additional file 2.

### Establishment of prognostic models

Three predictive models were established each for PFS and OS: a conventional model, an inter-tumor heterogeneity model, and an integrated model. The conventional model was established using conventional data, including age, CA125 level, FIGO stage, volume of ascites, characteristics of ascites, Sur_status, pixel number, MajorAxis, MinorAxis, average CT value, lymph node metastasis (LNM) location (pelvic, middle abdominal, upper abdominal, and distant LNM), number of metastatic implants, location of metastatic implants, pattern of invasion, ratio of solid components in the primary lesion, SUVmax, SUVmean, SUVpeak, and TLG. The inter-tumor heterogeneity model included the heterogeneity metrics calculated from both conventional measurements and texture features of the lesions. The integrated model included all the above-mentioned data. Finally, 6 models were established.

### Immunohistochemical scores of p53 and Ki-67

Postoperative pathological specimens of primary lesions were prepared by the pathology department of our hospital. All immunohistochemical sections obtained for p53 and Ki-67 measurements were scanned using the Pannoramic MIDI scanner (3DHISTECH, Budapest, Hungary) to generate digital Whole Slide Images (WSIs). The WSIs were then input into the Aipathwell software (Servicebio, Wuhan, China), which uses the deep learning principle of artificial intelligence to automatically analyze and calculate the histochemical scores (H-score) of p53 and Ki-67. The H-score was calculated as ([{% of weak staining} × 1] + [{% of moderate staining} × 2] + [{% of strong staining} × 3]) [33, 34].

### Statistical methods

The Shapiro–Wilk test was used to assess normal distribution. Normally distributed data were expressed as mean ± standard deviation (x ± s). Non-normally distributed data were expressed as median (upper quartile and lower quartile, represented as 1st Qu. to 3rd Qu.). Student’s t-test or Mann–Whitney *U* test was used to compare continuous variables of two groups, depending on the normality of the distribution. Enumeration data were analyzed by the chi-square test. The intraclass correlation coefficient (ICC) [35] was computed to assess the intra- and interobserver measurements.

**Fig. 1:**
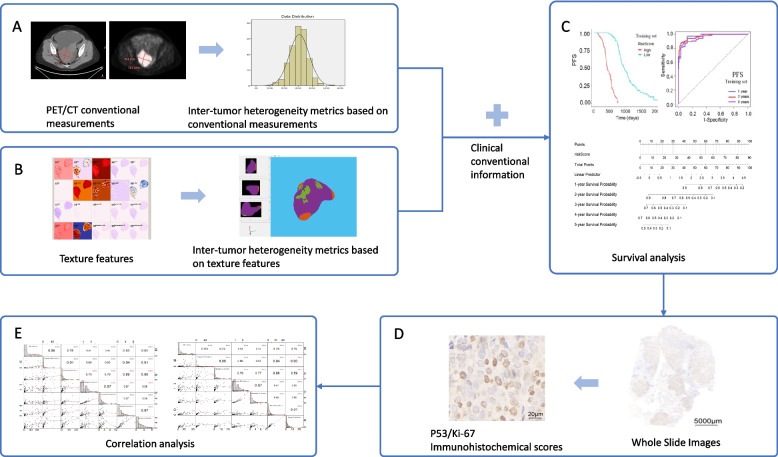
Data analysis workflow. **A** Extraction of inter-tumor heterogeneity metrics based on conventional measurements. **B** Extraction of inter-tumor heterogeneity metrics based on texture features. **C** Survival analysis using the prognostic model. **D** Immunohistochemical scores of p53 and Ki-67 using Whole Slide Images. **E** Correlation analysis between the immunohistochemical scores and the risk scores obtained from the models

Feature selection and modeling for survival analysis were performed using the least absolute shrinkage and selection operator (LASSO) [36] Cox regression method. The included patients from our larger affiliated hospital served as the training set and the patients from the other affiliated hospital served as the testing set. The model training uses fivefold cross-validation in training set. The Kaplan–Meier survival curve and log-rank test [37] were used for confirmation. Spearman’s correlation coefficient (ρ) was used to evaluate the correlation between the immunohistochemical scores and risk scores obtained from models. Texture features were computed using the Computational Environment for Radiological Research (CERR) software (https://github.com/cerr/CERR/) [38], which is IBSI compliant. Inter-tumor heterogeneity metrics based on texture features were extracted using MATLAB R2022a software (MathWorks, Natick, USA). Other statistical methods were conducted with R software (4.1.0, R Core Team). The main packages included in the study were “survival,” “survivalROC,” “survcomp,” “glmnet,” “ggplot2,” “pROC,” and “corrplot.” A *p*-value < 0.05 (two-sided) was considered statistically significant. The workflow of data analysis is shown in Fig. 1.

## Results

### Basic patient information

**Fig. 2:**
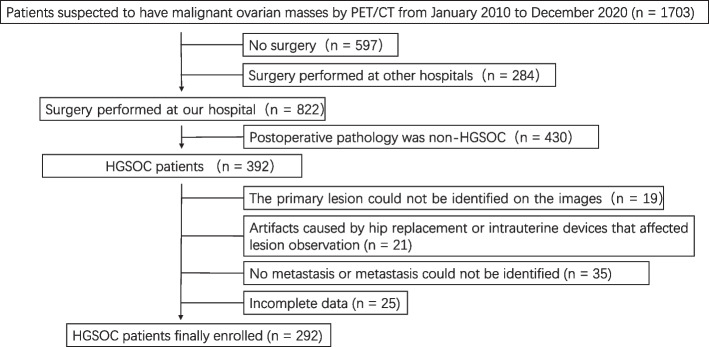
Flowchart of the enrolled patients

A total of 1749 patients with suspected malignant ovarian mass were retrospectively enrolled. Of these, 292 patients with HGSOC were finally included in the study. Figure 2 shows the flowchart of patients enrolled in the study cohort. The median age of the enrolled patients was 54 years (1st Qu.: 47 to 3rd Qu.: 61). The median follow-up time of all patients was 1021.5 days (1st Qu.: 717.7 days to 3rd Qu.: 1626.0 days). Patient characteristics for the two datasets are summarized in Tables 1 and 2.

**Table 1:** Clinical characteristics of the training set and the testing set

	**Training set (** ***n* = 208)**	**Testing set (** ***n*** **= 84)**	***p*** **-value** ^**a**^
**Age:median (1st Qu., 3rd Qu.)**	54.00(47.25,60.00)	54.50(47.00,64.00)	0.49
**CA125:median (1st Qu., 3rd Qu.)**	867.00(511.00,1717.75)	867.00(319.75,1787.75)	0.30
**Surgical resection status**			0.65
R0 (no residual tumor)	119(57.21%)	50(59.52%)	
R1 (residual tumor ≤ 1 cm)	42(20.19%)	19(22.62%)	
R2 (residual tumor > 1 cm)	47(22.60%)	15(17.86%)	
**FIGO stage determined surgically (%)**			0.93
IIB	3(1.44%)	1(1.19%)	
IIIA	6(2.88%)	1(1.19%)	
IIIB	20(9.62%)	9(10.71%)	
IIIC	119(57.21%)	45(53.57%)	
IVA	33(15.97%)	16(19.05%)	
IVB	27(12.98%)	12(14.29%)	
**Ascites volume**	1500.00(300.00,3000.00)	1000.00(200.00,3750.00)	0.50
**Ascites_character**			0.38
No ascites	9(4.33%)	6(7.14%)	
Bloody	63(30.29%)	20(23.81%)	
Non-bloody	136(65.38%)	58(69.05%)	
**Survival time:median (1st Qu., 3rd Qu.)**			
PFS	644.50(413.75,902.50)	657.00(422.00,902.00)	0.79
OS	1021.50(724.25,1630.00)	989.50(657.50,1729.75)	0.44

*Abbreviations: CA125* Cancer Antigen 125, *1st Qu*. First or upper quartile, *3rd Qu.* Third or lower quartile, *FIGO* International Federation of Gynecology and Obstetrics, *PFS* Progression-Free Survival, *OS* Overall Survival

^a^Differences in clinical characteristics were compared using Student’s t-test or Mann–Whitney *U* test or chi-square test

**Table 2:** Characteristics of lesions in the training set and the testing set

	**Training set (** ***n* = 208)**	**Testing set (** ***n* = 84)**	***p*-value**^a^
**Lymph node metastasis (LNM)**			
Pelvic LNM	74(35.58%)	25(29.76%)	0.34
Middle abdominal LNM	63(30.29%)	35(41.67%)	0.06
Upper abdominal LNM	61(29.33%)	30(35.71%)	0.29
Distant LNM	26(12.50%)	16(19.05%)	0.15
**Number of metastatic implants**	6.00(3.00,8.00)	5.50(3.25,8.00)	0.14
**Location of metastatic implants**			
AR-5 (left lower)	142(68.27%)	46(54.76%)	0.04*
AR-6 (pelvis)	173(83.17%)	75(89.29%)	0.19
AR-7 (right lower)	131(62.98%)	47(55.95%)	0.27
AR-4 (left flank)	88(42.31%)	35(41.67%)	0.92
AR-0 (central)	95(45.67%)	37(44.05%)	0.80
AR-8 (right flank)	147(70.67%)	55(65.48%)	0.38
AR-3 (left upper)	129(62.02%)	57(67.86%)	0.42
AR-2 (epigastrium)	87(41.83%)	41(48.81%)	0.28
AR-1 (right upper)	141(67.79%)	61(72.62%)	0.42
**Pattern of invasion**			0.74
0 No invasion	23(11.06%)	13(15.48%)	
1 Nodular type	42(20.19%)	18(21.43%)	
2 Predominantly nodular type	51(24.52%)	18(21.43%)	
3 Predominantly infiltrate type	52(25.00%)	17(20.24%)	
4 Infiltrate type	40(19.23%)	18(21.43%)	

*Abbreviations: LNM* Lymph Node Metastasis, *AR* area

^a^Differences in clinical characteristics were compared using Student’s t-test or Mann–Whitney *U* test or chi-square test. “*” represents statistically significant

### Establishment of survival predictive models

**Fig. 3:**
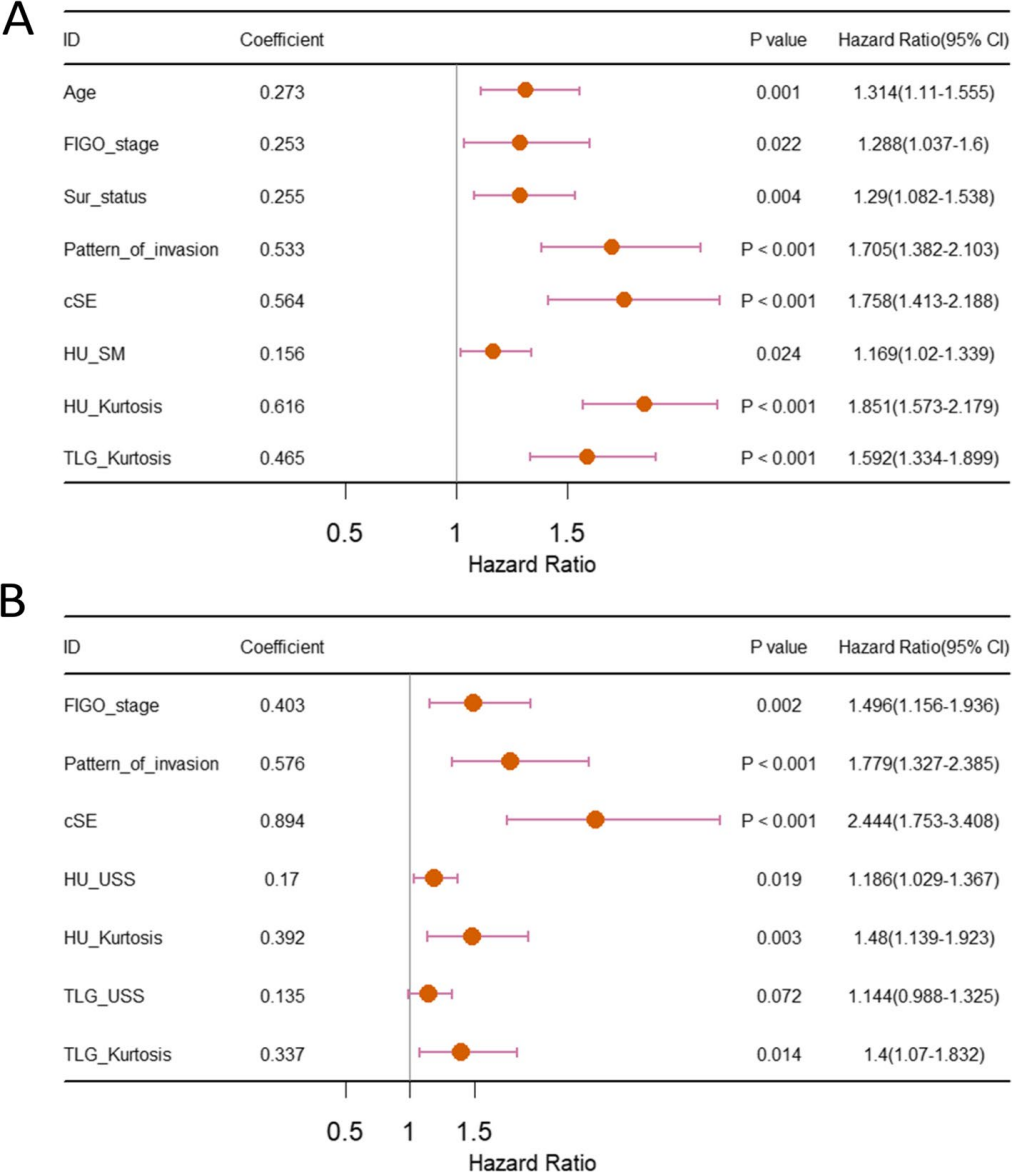
Parameters of the models. The related features in the integrated PFS model (**A**) and integrated OS model (**B**). FIGO_stage: the International Federation of Gynecology and Obstetrics stage; Sur_status: surgical excision status; cSE: cluster site entropy; HU_SM: standard error of CT value; HU-Kurtosis: kurtosis value of CT value; TLG-Kurtosis: kurtosis value of the total amount of glucose decomposition; HU_USS: uncorrected sum of squares of CT value; TLG_USS: uncorrected sum of squares of total lesion glycolysis

**Fig. 4:**
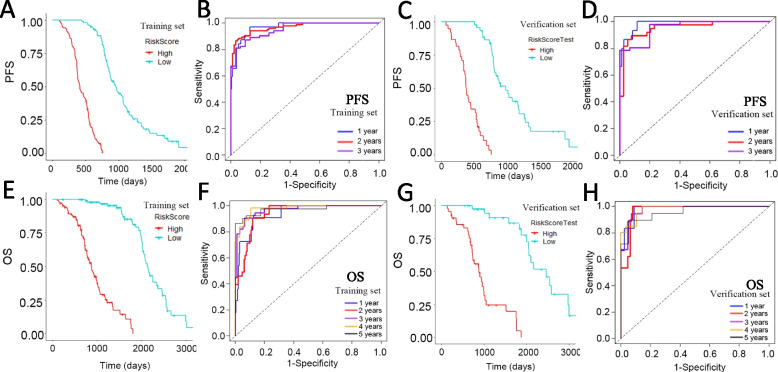
Kaplan–Meier survival curves and ROC curves of the integrated models: 1- to 3-year progression-free rate in the training set (**A**, **B**) and the testing set (**C, D**); 1- to 5-year survival rate in the training set (**E**, **F**) and the testing set (**G**, **H**)

**Fig. 5:**
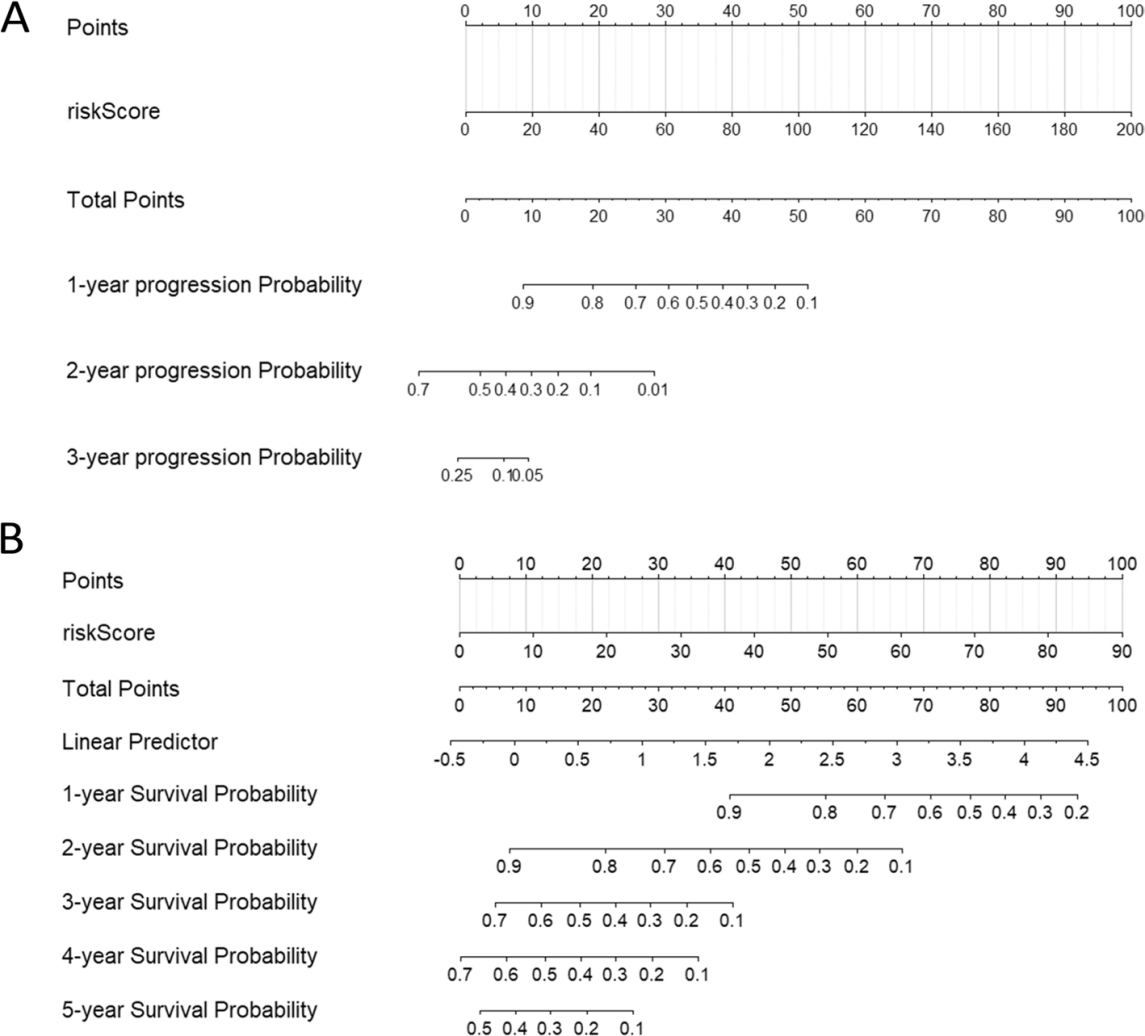
Nomogram of the integrated PFS predictive model (**A**) and the integrated OS predictive model (**B**)

A total of 107 inter-tumor heterogeneity indicators were extracted from conventional measurements (*n* = 104) and Haralick texture features (*n* = 3). The intra- and interobserver ICCs of heterogeneity indicators were greater than 0.75. Table 3 shows a comparison of the predictive performance of the models. The C-indices of the integrated model for both PFS and OS were the highest. The C-indices of the training and testing set for the integrated PFS model were 0.898 (95% confidence interval [CI]: 0.881–0.914) and 0.891 (95% CI: 0.860–0.921), respectively. The C-indices of the training set and the validation set of the integrated OS model were 0.894 (95% CI: 0.871–0.917) and 0.905 (95% CI: 0.873–0.936), respectively. These parameters are shown in Figs. 3 and 4. Figure 5 shows the nomogram of the integrated PFS and OS predictive models. The inter-tumor risk scores calculated from the inter-tumor heterogeneity model were significant prognostic factors for PFS (hazard ratio [HR] = 1.281, 95% CI: 1.240–1.322, *p* < 0.05) and OS (HR = 1.221, 95% CI: 1.174–1.270, *p* < 0.05) by univariate Cox analysis. Multivariate Cox analysis also indicated that the inter-tumor risk scores were independent prognostic factors for PFS (HR = 1.214, 95% CI: 1.170–1.260, *p* < 0.05) and OS (HR = 1.187, 95% CI: 1.154–1.221, *p* < 0.05).

**Table 3:** Comparison of the predictive performance of the different models

	**Training set (95% CI)**	**Testing set (95% CI)**
**PFS predictive models (C-index)**		
Conventional model	0.809(0.778–0.835)	0.808(0.766–0.851)
Inter-tumor heterogeneity model	0.853(0.816–0.870)	0.849(0.807–0.891)
Integrated model	0.898(0.881–0.914)	0.891(0.860–0.921)
**OS predictive models (C-index)**		
Conventional model	0.821(0.751–0.840)	0.817(0.758–0.877)
Inter-tumor heterogeneity model	0.854(0.812–0.876)	0.852(0.788–0.916)
Integrated model	0.894(0.871–0.917)	0.905(0.873–0.936)

*Abbreviations*: *PFS* Progression-Free Survival, *OS* Overall Survival, *CI* Confidence Interval, *C-index* Concordance Index

### Correlation analysis between model risk scores and immunohistochemical scores of p53 and Ki-67

**Fig. 6:**
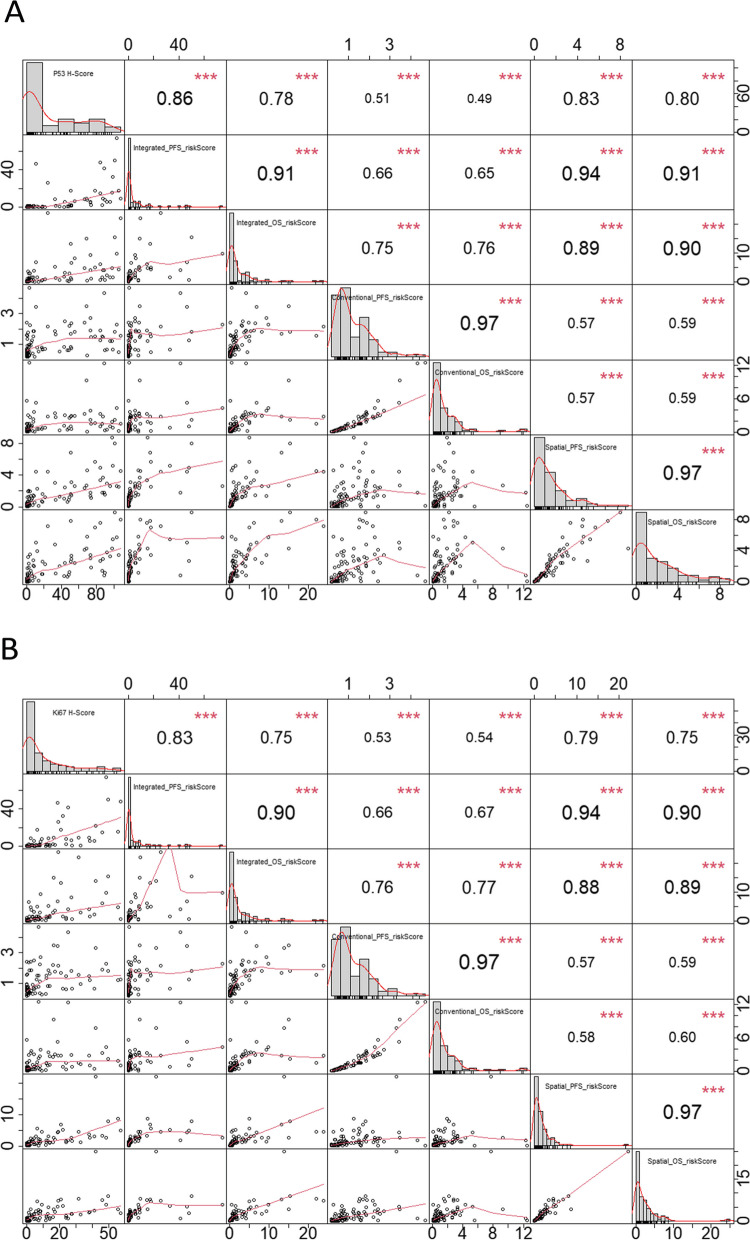
Spearman’s correlation coefficient graph. The correlation between the risk scores of the prognostic models and immunohistochemical scores of p53 (**A**) and Ki-67 (**B**). The distribution of each variable is shown on the diagonal line, including p53/Ki-67 H-Score, Integrated_PFS_riskScore, Integrated_OS_riskScore, Conventional_PFS_riskScore, Conventional_OS_riskScore, Inter-tumor_PFS_riskScore, and Inter-tumor_OS_riskScore. The part below the diagonal line shows the scatterplots and fitting curves of the two variables. The part above the diagonal line shows Spearman’s correlation values of the two variables and the corresponding significance levels: *** represents *p* < 0.001

Immunohistochemical analysis for p53 expression was performed for 90 of 292 patients. The p53 H-score showed the strongest correlation with the risk score of the integrated PFS predictive model (*ρ* = 0.859, *p* < 0.001) (Fig. 6A). Immunohistochemical analysis for Ki-67 expression was performed for 88 of 292 patients. The Ki-67 H-score showed the strongest correlation with the risk score of the integrated PFS predictive model (*ρ* = 0.829, *p* < 0.001) (Fig. 6B).

## Discussion

In the present study, we extracted qualitative inter-tumor heterogeneity metrics from conventional measurements and texture features from ^18^F-FDG PET/CT images. The models based on these metrics performed well in predicting PFS and OS in patients with HGSOC. The risk scores derived from the models showed a relationship with p53 and Ki-67 expression levels.

The inter-tumor risk score recalculated from inter-tumor heterogeneity metrics was a strong and independent prognostic factor for PFS and OS. For conventional measurements, the results indicated that the more discrete the conventional measurements, the stronger the heterogeneity between tumor sites, and the shorter the PFS and OS for HGSOC patients. To date, few studies have focused on predicting the prognosis of patients with ovarian cancer by using heterogeneity metrics derived from conventional measurements. Lee et al. reported that the intra-tumor heterogeneity of ^18^F-FDG uptake on ^18^F-FDG PET/CT was significantly correlated with the recurrence of epithelial ovarian cancer [39]. Liu et al. showed that the SUVmean of primary tumors was higher than that of metastatic implants in the omentum [40]. For inter-tumor heterogeneity metrics based on texture features, cSE and cluDiss were important risk factors for PFS and OS; this finding is consistent with those of previous studies [15, 16, 41]. Both the cSE and cluDiss can be used as indicators of the extent of dissimilarity. Thus, patients with VOIs with highly similar textures will have low cSE and cluDiss values, implying low heterogeneity.

Image-based inter-tumor heterogeneity may be associated with underlying molecular changes. This study confirmed that the risk scores of the prognostic models based on inter-tumor heterogeneity metrics were strongly correlated with the expression of p53 and Ki-67. p53 expression reflects the TP53 missense mutation, and it plays an important role in regulating cell proliferation, apoptosis, senescence, DNA repair, and metabolic homeostasis [42–44]. Ki-67 is closely associated with tumor differentiation, invasion, metastasis, and prognosis [45, 46]. According to previous studies, CT-based inter-tumor heterogeneity metrics were correlated with the enrichment of the WNT/ β-catenin signaling pathway [15], 19q12 amplification involving CCNE1 [41], and abundance of some proteins in vivo [47].

However, surgical procedures still play a major role in the primary treatment of ovarian cancer, and immunohistochemical determination of the surgically removed tissue is usually feasible. However, some patients, particularly those with advanced ovarian cancer, require neoadjuvant chemotherapy, rather than direct surgery. Therefore, it is necessary to analyze the correlation between noninvasive indicators and these immunohistochemical indicators to identify the potential molecular mechanisms underlying inter-tumor heterogeneity. Moreover, expanding their scope of application, such as predicting the survival of patients using neoadjuvant chemotherapy, evaluating the efficacy of neoadjuvant chemotherapy, and developing personalized treatment plans, is crucial [48].

Through this study, we hope to preliminarily establish inter-tumor heterogeneity metrics based on ^18^F-FDG PET/CT and identify high-risk patients with high heterogeneity. Greater focus should be directed toward these patients, and more proactive personalized treatment plans could be developed to improve patient management. This study provides a nomogram for clinical use. In the future, we hope to standardize and simplify this process. First, we will use a standardized ^18^F-FDG PET/CT scanning process within 15 days before surgery. Second, by combining information from the hospital information system and Picture Archiving and Communication System, the integrated software can automatically calculate inter-tumor heterogeneity metrics and provide the risk level, possible prognosis, and available personalized treatment plans.

It is difficult to distinguish metastatic malignant lesions from inflammatory sites using ^18^F-FDG PET/CT images [49], especially for inexperienced readers, and relevant patient history and symptoms, knowledge of the typical pattern of metastases for the malignancy under investigation, corresponding CT images, and the help of clinical doctors may guide the interpretation of ^18^F-FDG uptake [50]. Furthermore, this study used a 9-zone abdominal location method to locate metastatic sites, which is relatively simple and user-friendly, particularly for novices.

The present study had some limitations. First, the study cohort was limited in size. However, as ^18^F-FDG PET/CT technology continues to advance and become more accessible, a larger number of patients will likely have the opportunity to undergo ^18^F-FDG PET/CT examinations for disease evaluation. Second, we used traditional artificial segmentation for delineating VOI. Although it was drawn and corrected by two experienced imaging radiologists, artificial errors were unavoidable. Third, when constructing the PFS and OS models, we did not include the interaction between variables in the models; however, this also increased the simplicity and interpretability of the models. Fourth, it is important to acknowledge that this study specifically pertains to patients with at least one metastatic lesion and may not be applicable to all types of HGSOC.

## Conclusions

In conclusion, inter-tumor heterogeneity metrics based on two dimensions (conventional measurements and texture features) of two modalities (PET and CT) in ^18^F-FDG PET/CT were developed and used to construct noninvasive predictive models of PFS and OS for patients with HGSOC. These models need to be validated in larger multicenter cohorts and are expected to be implemented in clinical practice in the future. The improved inter-tumor heterogeneity extraction method is also expected to be applied to other tumors with inter-tumor heterogeneity, thus providing a new research approach.

### Supplementary Information

**Supplementary Material 1.**

**Supplementary Material 2.**

## Data Availability

The datasets used and/or analyzed during the current study are available from the corresponding author on reasonable request.

## Abbreviations

HGSOC: High-grade serous ovarian cancer
^18^F-FDG PET/CT: ^18^F-fluoro-2-deoxyglucose Positron Emission Tomography/Computed Tomography
FIGO: International Federation of Gynecology and Obstetrics
PFS: Progression free survival
OS: Overall survival
VOI: Volume of interest
LASSO: Least absolute shrinkage and selection operator
ROC: Receiver operating characteristic
AUC: Area under the curve
cSE: Cluster sites entropy
cluDev: Cluster standard deviation
cluDiss: Cluster dissimilarity
WSI: Whole Slide Image
