# Supplementary material for: Survival time prediction in patients with high-grade serous ovarian cancer based on 18F-FDG PET/CT- derived inter-tumor heterogeneity metrics
Source: BMC Cancer. 2024 Mar 12;24:337. doi: 10.1186/s12885-024-12087-y (PMC10936071; doi:10.1186/s12885-024-12087-y)
Supplement: Supplementary file 1 — Supplementary Material 1. [file 12885_2024_12087_MOESM1_ESM.docx]

**Additional file 1**

**Abdominopelvic region assessment method for encoding lesion sites**

The abdominopelvic 9-zone method was used to encode all lesion sites, including primary lesion and peritoneal implants [1], as shown in Fig. S1. The abdomen is divided into 9 regions by two transverse planes and two sagittal planes. The upper and lower transverse planes are the lowest aspect of the costal margin and the anterior superior iliac spine. The two sagittal planes divide the abdominopelvic cavity into three equal parts. The umbilical region is designated AR0. Starting from the right upper part to the right middle part, the regions are numbered AR1-AR8 clockwise [1-3]. The anatomical structures included in each region are shown in Table S1.

**
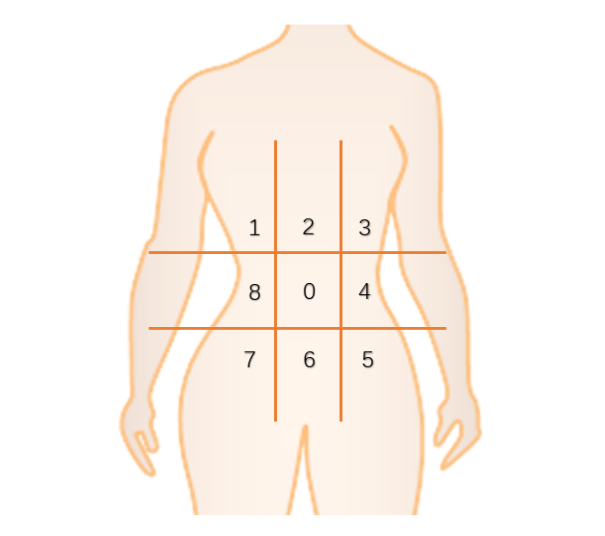
**

**Fig. S1** Abdominopelvic region assessment.

**Table S1. Anatomical structures included in each region**

| Regions | Anatomic structures included |
| --- | --- |
| AR-0 | midline abdominal incision, the greater omentum and the transverse colon |
| AR-1 | superior surface of the right lobe of the liver and the undersurface of the right hemidiaphragm. |
| AR-2 | the epigastric fat pad, the left lobe of the liver, the lesser omentum, and the falciform ligament |
| AR-3 | the undersurface of the left hemidiaphragm, the spleen, the tail of the pancreas, and the anterior and posterior surfaces of the stomach |
| AR-4 | the descending colon and the left abdominal gutter |
| AR-5 | the pelvic side wall lateral to the sigmoid colon and the sigmoid colon itself |
| AR-6 | the female internal genitalia with ovaries, tubes, and uterus; bladder; cul-de-sac of Douglas; and the rectosigmoid colon |
| AR-7 | the right pelvic side wall and the base of the cecum, including the appendix |
| AR-8 | the right abdominal gutter and the ascending colon |

Abbreviations: AR: area.

**References**

1. Diaz-Gil D, Fintelmann FJ, Molaei S, Elmi A, Hedgire SS, Harisinghani MG: **Prediction of 5-year survival in advanced-stage ovarian cancer patients based on computed tomography peritoneal carcinomatosis index**. *Abdominal Radiology* 2016, **41**(11):2196-2202.

2. Lin CN, Huang WS, Huang TH, Chen CY, Huang CY, Wang TY, Liao YS, Lee LW: **Adding Value of MRI over CT in Predicting Peritoneal Cancer Index and Completeness of Cytoreduction**. *Diagnostics (Basel)* 2021, **11**(4).

3. Jacquet P, Sugarbaker PH: **Clinical research methodologies in diagnosis and staging of patients with peritoneal carcinomatosis**. *Cancer Treat Res* 1996, **82**:359-374.
