# Supplementary material for: Survival time prediction in patients with high-grade serous ovarian cancer based on 18F-FDG PET/CT- derived inter-tumor heterogeneity metrics
Source: BMC Cancer. 2024 Mar 12;24:337. doi: 10.1186/s12885-024-12087-y (PMC10936071; doi:10.1186/s12885-024-12087-y)
Supplement: Supplementary file 2 — Supplementary Material 2. [file 12885_2024_12087_MOESM2_ESM.docx]

**Additional file 2**

**Extraction of inter-tumor heterogeneity metrics based on texture features**

The computed tomography (CT) images and corresponding volumes of interest (VOIs) were imported into MATLAB R2022a (MathWorks, Natick, MA, USA). An in-house MATLAB program was used to extract inter-tumor heterogeneity metrics based on texture features. The relevant functions in the Computational Environment for Radiological Research (CERR) software (https://github.com/cerr/CERR/) package were used to calculate texture feature parameters [1]. CERR is a comprehensive and open-source MATLAB platform used for reproducible radiomics research. The radiomic texture features in CERR were defined in accordance with the Image Biomarker Standardization Initiative (IBSI) guidelines. Similar voxels were subsequently classified into clusters based on the similarity of their textural features. The parameters calculated based on the clustering results were further extracted to reflect inter-tumor heterogeneity. These steps are described in detail below:

1. First, the CT images were rescaled and 256 gray levels were used. Moreover, a bin width of 32 was used to discretize the images. The patch-wise Gray-Level Co-occurrence Matrix (GLCM) of each VOI voxel was calculated with a set patch size of 5 × 5 × 1[1]. The Haralick texture features for each voxel in the VOIs, including energy, entropy, contrast, and homogeneity, were calculated using the CERR functions, which complied with IBSI [2].
2. Based on the above four Haralick texture features, the voxels in all VOIs of each patient were clustered using Gaussian Mixture Models (GMM) [3]. The iterative Expectation-Maximization (EM) algorithm was used to fit GMMs to the data [4]. The number of clusters was set from two to five categories, and the corresponding Bayes Information Criterion (BIC) was calculated for the clustering results for each category [4]. The BIC is a model selection tool used to compare multiple models that fit to the same data. This is a likelihood-based measure of model fit that includes a penalty for complexity, specifically, the number of parameters. When comparing multiple models, the model with the lowest BIC value was considered the best fit. Thus, the number of corresponding clusters with the minimum BIC was determined, thereby dividing the voxels in the VOIs into a corresponding number of subclass groups, and these subclasses were used in the subsequent calculations. Each subclass was described using the collection of the mean values of Haralick textures.
3. Subsequently, the textural dissimilarities between pairs of all subclasses were calculated using Euclidean distance [5]. A dissimilarity matrix was then obtained. This matrix reflects the heterogeneity information between all subclasses. To maintain the comparability and robustness of calculation results between different patients, the matrix mentioned above was discretized to 10 bins. Thus, the dissimilarity matrix D was obtained. The z-score of the dissimilarity results was normalized, and the tumor heterogeneity metric, cluster site entropy (cSE), was calculated based on the entropy of matrix D using the Shannon entropy formulation. The subclasses with highly distinct textures were less similar and had higher cSE values. The formula for this calculation is as follows [6]:

$$cSE=-\frac{1}{N}\sum_{n=1}^{N} p(d_{n})\log_{2} \left( p\left( d_{n} \right) \right) (1.1)$$

where *N* is the number of bins (*N* = 10) and *p (d)* corresponds to the normalized dissimilarities *d*.

1. The grey-level distance zone matrix (GLDZM) was calculated for the matrix D [7]. GLDZM is a two-dimensional histogram that captures the prevalence of dissimilarities shared by different numbers of subclasses. The elements of the GLDZM are the number of discrete dissimilarities and the number of subclass pairs sharing these dissimilarities (Fig. S2). The GLDZM column corresponds to the level of discrete dissimilarities, while the GLDZM row corresponds to the number of subclass pairs that share these dissimilarities. The arrangement of the GLDZM started from the primary lesion, and each metastatic lesion was arranged in a counterclockwise direction. The arrangement of the lesion sites in each patient was consistent.


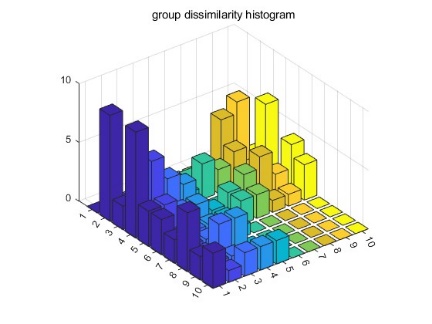


**Fig. S2** Two-dimensional histogram of the dissimilarity matrix

Two heterogeneity features, i.e., cluster standard deviation (cluDev) and cluster diversity (cluDiss), were calculated from the GLDZM. CluDev is the standard deviation of the GDM, and the formula for calculating cluDiss is as follows [6]:

$$\begin{aligned} cluDiss=\sqrt{\frac{1}{K\times M}\sum_{i}^{K} \sum_{j}^{M} \left( i+j-\hat{D}-\hat{A} \right)^{\alpha}\times G\left( i,j \right)}\#\left( 1.2 \right) \end{aligned}$$

where *K* is the number of dissimilarity levels, *M* is the size level of all groups, $\hat{D}$ is the normalized average of the dissimilarities, $\hat{A}$ is the normalized average of group size, *G* is the dissimilarity matrix GDM, and *α* = 4. The indices *i* and *j* correspond to the dissimilarity and size of the group, respectively.

**References**

1. Apte AP, Iyer A, Crispin-Ortuzar M, Pandya R, van Dijk LV, Spezi E, Thor M, Um H, Veeraraghavan H, Oh JH *et al*: **Technical Note: Extension of CERR for computational radiomics: A comprehensive MATLAB platform for reproducible radiomics research**. *Med Phys* 2018.

2. Vargas HA, Veeraraghavan H, Micco M, Nougaret S, Lakhman Y, Meier AA, Sosa R, Soslow RA, Levine DA, Weigelt B *et al*: **A novel representation of inter-site tumour heterogeneity from pre-treatment computed tomography textures classifies ovarian cancers by clinical outcome**. *Eur Radiol* 2017, **27**(9):3991-4001.

3. Reynolds D: **Gaussian mixture models**. *Encyclopedia of biometrics* 2009, **741**:659-663.

4. Neath AA, Cavanaugh JE: **The Bayesian information criterion: background, derivation, and applications**. *WIREs Computational Statistics* 2011, **4**(2):199-203.

5. Veeraraghavan H, Vargas HA, Sanchez AJ, Micco M, Mema E, Lakhman Y, Crispin-Ortuzar M, Huang EP, Levine DA, Grisham RN *et al*: **Integrated Multi-Tumor Radio-Genomic Marker of Outcomes in Patients with High Serous Ovarian Carcinoma**. *Cancers (Basel)* 2020, **12**(11).

6. Himoto Y, Veeraraghavan H, Zheng J, Zamarin D, Snyder A, Capanu M, Nougaret S, Vargas HA, Shitano F, Callahan M *et al*: **Computed Tomography–Derived Radiomic Metrics Can Identify Responders to Immunotherapy in Ovarian Cancer**. *JCO Precision Oncology* 2019(3):1-13.

7. Thibault G, Angulo J, Meyer F: **Advanced Statistical Matrices for Texture Characterization: Application to Cell Classification**. *IEEE Transactions on Biomedical Engineering* 2014, **61**(3):630-637.
